# Glycomacropeptide for Management of Insulin Resistance and Liver Metabolic Perturbations

**DOI:** 10.3390/biomedicines9091140

**Published:** 2021-09-02

**Authors:** Mathilde Foisy Sauvé, Francis Feldman, Mireille Koudoufio, Nour-El-Houda Ould-Chikh, Lena Ahmarani, Alain Sane, Thierry N’Timbane, Ramy El-Jalbout, Nathalie Patey, Schohraya Spahis, Alain Stintzi, Edgard Delvin, Emile Levy

**Affiliations:** 1 Research Center, CHU Ste-Justine, Montréal, QC H3T 1C5, Canada; mathilde.foisy.sauve@umontreal.ca (M.F.S.); francis.feldman@umontreal.ca (F.F.); mireille.koudoufio@umontreal.ca (M.K.); nour-el-houda.ould-chikh@umontreal.ca (N.-E.-H.O.-C.); lena.ahmarani@gmail.com (L.A.); sanealaintheo@gmail.com (A.S.); mintyathierry@yahoo.ca (T.N.); ramy.el-jalbout.med@ssss.gouv.qc.ca (R.E.-J.); natalie.patey.hsj@ssss.gouv.qc.ca (N.P.); schohraya.spahis.hsj@ssss.gouv.qc.ca (S.S.); delvine@sympatico.ca (E.D.); 2 Department of Nutrition, Université de Montréal, Montréal, QC H3C 3J7, Canada; 3 Department of Radiology, Université de Montréal, Montréal, QC H3T 1C5, Canada; 4 Department of Pathology, Université de Montréal, Montréal, QC H3T 1C5, Canada; 5 Department of Biochemistry, Microbiology, and Immunology, Faculty of Medicine, Ottawa Institute of Systems Biology, University of Ottawa, Ottawa, ON K1H 8M5, Canada; astintzi@uottawa.ca; 6 Department of Biochemistry, Université de Montréal, Montréal, QC H3T 1C5, Canada

**Keywords:** milk peptide, nutraceutical, metabolic syndrome, metabolism, insulin signaling, inflammation, oxidative stress, mice, mitochondria dysfunction

## Abstract

Background and Aims: The increasing prevalence and absence of effective global treatment for metabolic syndrome (MetS) are alarming given the potential progression to severe non-communicable disorders such as type 2 diabetes and nonalcoholic fatty liver disease. The purpose of this study was to investigate the regulatory role of glycomacropeptide (GMP), a powerful milk peptide, in insulin resistance and liver dysmetabolism, two central MetS conditions. Materials and Methods: C57BL/6 male mice were fed a chow (Ctrl), high-fat, high-sucrose (HFHS) diet or HFHS diet along with GMP (200 mg/kg/day) administered by gavage for 12 weeks. Results: GMP lowered plasma insulin levels (in response to oral glucose tolerance test) and HOMA-IR index, indicating a more elevated systemic insulin sensitivity. GMP was also able to decrease oxidative stress and inflammation in the circulation as reflected by the decline of malondialdehyde, F2 isoprostanes and lipopolysaccharide. In the liver, GMP raised the protein expression of the endogenous anti-oxidative enzyme GPx involving the NRF2 signaling pathway. Moreover, the administration of GMP reduced the gene expression of hepatic pro-inflammatory COX-2, TNF-α and IL-6 via inactivation of the TLR4/NF-κB signaling pathway. Finally, GMP improved hepatic insulin sensitization given the modulation of AKT, p38 MAPK and SAPK/JNK activities, thereby restoring liver homeostasis as revealed by enhanced fatty acid β-oxidation, reduced lipogenesis and gluconeogenesis. Conclusions: Our study provides evidence that GMP represents a promising dietary nutraceutical in view of its beneficial regulation of systemic insulin resistance and hepatic insulin signaling pathway, likely via its powerful antioxidant and anti-inflammatory properties.

## 1. Introduction

The impact of bovine milk consumption on human health has always sparked fierce debate, which has prompted many scientists to reassess its attributes [1,2]. On the one hand, the quality of lipids in milk has been criticized given the negative impact of saturated fats on cardiovascular health [3,4], and the possible contribution of its high energy content to obesity. On the other hand, epidemiologic studies have stressed the association of bovine milk consumption with the reduced risk of developing obesity, metabolic syndrome (MetS), diabetes and cardiovascular diseases [2,5]. Currently, there is a renewed interest in valuable bioactive peptides derived from major human and bovine milk proteins given their high potential in magnifying health benefits for neonates, infants, adolescents and adults [6,7]. Especially, they exert remarkable protective effects on cardiometabolic health [6,8]. For example, lactoferrin has demonstrated powerful antioxidant and anti-inflammatory activities [9], whereas β-lactoglobulin has been found to possess antihypertensive properties [8].

Glycomacropeptide (GMP) is another natural bioactive milk-derived compound that has attracted scientist attention in view of its numerous biological activities [10]. GMP was first studied because of its unique amino acid profile, which lacks phenylalanine, tyrosine and tryptophan, making it an excellent source of low-phenylalanine protein for individuals diagnosed with phenylketonuria [11]. Subsequently, GMP showed ability to: (i) regulate proliferation and intracellular lipid accumulation in vitro [12]; (ii) modulate adipose tissue metabolism [13]; (iii) bind enterotoxins, inhibit bacterial and viral adhesion [14]; (iv) promote bifidobacterial growth; and (v) influence immune system responses [15]. Recently, our group revealed GMP ability to exert powerful antioxidative and anti-inflammatory activities in intestinal Caco-2/15 cells, while pointing out its significant impact on insulin signaling and lipoprotein production, suggesting a potential role in the alleviation of MetS [16]. MetS represents a cluster of interrelated risk factors, including abdominal obesity, dyslipidemia, hypertension and insulin resistance (IR)-mediated hyperglycemia [17]. Redox imbalance and inflammation are considered as key contributors to IR and MetS pathogenesis [18,19]. As there is so far no effective unifying treatment for patient management, MetS may progress to diabetes and cardiovascular diseases [20]. Furthermore, side effects of the current pharmacologic drugs are significant, and still far from ideal, which better favors therapeutic nutritional avenues. It is particularly important to note the close relationship between MetS and nonalcoholic fatty liver disease (NAFLD), the most common liver disorder with a marked prevalence worldwide [21]. NAFLD may progress from hepatic steatosis without liver injury to fibrosis and cirrhosis [22]. Independently from the current debate whether NALFD is a consequence of MetS or whether NAFLD is an etiological factor for MetS and diabetes [23], the bidirectional nature of their relationship would benefit from the development of effective nutritional drugs.

Although in vitro and in vivo investigations suggest the ability of GMP to alleviate cardiometabolic disorders, the evidence is uneven regarding its effects on MetS components, such as obesity [24], glucose metabolism abnormalities and IR [25,26], dyslipidemia [13] and hypertension [24]. Therefore, additional efforts should be deployed before drawing final conclusion on GMP effectiveness. To participate in the general endeavor, the present study was designed to examine whether GMP can provide an ameliorating effect on C57BL/6 mice maintained on an obesogenic high-fat, high-sucrose diet (HFHS).

## 2. Materials and Methods

### 2.1. Glycomacropeptide

Purified GMP was obtained from Agropur Dairy Cooperative (Eden Prairie, MN, USA).

### 2.2. Animals

Eight-week-old C57BL/6 male mice (*n* = 36) were purchased from Charles River (Montreal, QC, Canada) and housed in a controlled environment (temperature of 22 ± 1 °C, 12-h daylight cycle) with free access to food and water. During the first week, mice were acclimatized with a chow diet. Thereafter, mice were separated in individual cages and randomly assigned to one of the dietary conditions for 12 weeks: chow diet (Ctrl, *n* = 12) (18.6% protein, 44.2% carbohydrates and 6.2% fat; 3.1 kcal/g, Teklad 2018, Harlan Laboratories); high-fat, high sucrose diet (HFHS, *n* = 12) (15% protein, 20% sucrose and 65% fat; 5.2 kcal/g, Research Diets, New Brunswick, NJ, USA) and HFHS diet supplemented with GMP (HFHS+GMP, *n* = 12). GMP solution (200 mg/kg body weight) or vehicle (water) was administered daily by gavage. Body weight and food intake were recorded two and three times, respectively, per week. After 12 weeks of treatment, animals were anesthetized with a mixture of ketamine/xylazine/acepromazine (100/10/1 mg/kg) and euthanized by cardiac puncture. Blood was collected in EDTA coated tubes and plasma was separated from cells by centrifugation at 3000× *g* for 20 min at 4 °C. Organs such as liver were dissected, weighted and immersed in liquid nitrogen before long-term storage at −80 °C for further experiments. But before storage, tissue samples were either placed in TRIzol (Life technologies, Carlsbad, CA, USA) for mRNA measurements or fixed in 10% neutral buffered formalin for histological examination. All animal manipulations were approved by the Institutional Animal Care Committee of the Sainte-Justine UHC Research Center.

### 2.3. Glucose Homeostasis

At week 10, an oral glucose tolerance test (OGTT) was performed after mice were fasted overnight. Blood was collected before (0 min) and after (15, 30, 60, 90, and 120 min) glucose administration by gavage (1 g glucose/kg body weight) for glycemia and insulinemia determinations. HOMA-IR index was then calculated using the following formula: fasting insulinemia (mUI/mL) × fasting glycemia (mM)/22.5.

### 2.4. Biochemical and Lipid Analysis

Plasma insulin (Millipore, Burlington, MA, USA) and lipopolysaccharide (LPS, MyBioSource, San Diego, CA, USA) concentrations were measured using rat/mouse ELISA kits. F2-isoprostane levels were measured by extraction of isoprostanes from plasma samples, adding a mixture of F2 isoprostanes as internal standard (Concordia University, Montreal, QC, Canada) for mass spectrometric quantification [27,28]. Plasma levels of triglyceride (TG) and total cholesterol (TC) were assessed using commercial kits (Randox Laboratories, Crumlin, UK). Lipid peroxidation was estimated by measuring plasma malondialdehyde (MDA) concentration by HPLC as previously described [29].

### 2.5. Liver Histology

Liver tissue samples were fixed in 10% neutral buffered formalin, dehydrated in gradient ethanol series and embedded in paraffin. For histological evaluation, 3 μm-thick tissue sections were stained with haematoxylin-phloxine saffron and examined under an optic microscope. Images of stained tissues were captured by a Zeiss Imager A1 (Carl Zeiss, Jena, Germany). Measurements were taken with the axiovision software allowing quantification of both microvesicular and macrovesicular lipids droplets. Briefly, the presence of multiple vacuoles smaller than the central nucleus was defined as microvesicular steatosis, whereas the presence of a single large vacuole that is larger than the nucleus and usually displaces it to the periphery of the cell was defined as macrovesicular steatosis [30]. Percentages of microvesicular or macrovesicular steatosis were determined calculating the steatotic fraction area relative to the entire tissue area.

### 2.6. RNA Extraction and RT-qPCR Analysis

Hepatic samples were homogenized in TRIzol reagent and total RNA was extracted using Invitrogen PureLink RNA Mini Kit (ThermoFisher, Waltham, MA, USA). RNA concentration and purity were determined by a Biodrop Touch Duo spectrophotometer (Montreal Biotech Inc., Dorval, QC, Canada) [31]. Complementary DNA was obtained by reverse transcripting 1 μg of RNA with the Superscript VILO Master Mix (Invitrogen, Waltham, MA, USA). Gene expression was analyzed by quantitative RT-PCR using the 7500 Fast Real-Time PCR System (Applied Biosystems, Waltham, MA, USA). The thermal profile included an initial denaturation at 95 °C for 30 s, followed by 40 cycles of denaturation at 95 °C for 3s, and annealing and extension at 60 °C for 30 s. Amplified gene expressions were quantified by fluorescence using the PowerUp SYBR Green Master Mix (Life Technologies, Carlsbad, CA, USA) [31]. Levels of expression of target-gene mRNAs were calculated by the 2^−∆∆CT^ method. The list of all primers can be found in the Supplementary Materials (Table S1).

### 2.7. Western Blot Analysis

Liver tissues were prepared for Western blot as previously described [16,29,31]. Briefly, proteins were separated on a 10% SDS-PAGE gel and electroblotted onto nitrocellulose membranes. Nonspecific binding sites were blocked using defatted milk proteins and membranes were incubated overnight at 4 °C in blocking solution with the following primary antibodies: acetyl CoA carboxylase (ACC, 280 kDa, 1/1000) Cell Signaling, Danvers, MA, USA, AKT (60 kDa, 1/1000) Cell Signaling, fatty acid synthase (FAS, 273 kDa, 1/1000) Cell Signaling, phospho-p38 mitogen-activated protein kinases (p-p38 MAPK, 43 kDa, 1/1000) Cell Signaling, phospho stress-activated protein kinases/Jun amino-terminal kinases (p-SAPK/JNK, 46 kDa, 1/500) Cell signaling, SAPK/JNK (46 kDa, 1/1000) Cell Signaling; carnitine palmitoyl transferase 1A (CPT1A, 88 kDa, 1/1000) Cell Signaling, glyceraldehyde 3-phosphate dehydrogenase (GAPDH, 1/1000, 37 kDa) Invitrogen, superoxide dismutase 2 (SOD2, 21 kDa, 1/3000) Invitrogen; glutathione peroxidase 1 (GPx1, 26 kDa, 1/1000) Novus Biologicals, Centennial, CO, USA, interlukin-6 (IL-6, 24 kDa, 1/1000) Novus Biologicals; glucose-6-phosphatase (G-6-Pase, 1/1000, 58 kDa) Abcam, Cambridge, UK, nuclear factor erythroid-2-related factor 2 (NRF2, 110 kDa, 1/1000) Abcam, peroxisome proliferator activated receptor coactivator 1a (PGC-1α, 130 kDa, 1/1000) Abcam, phosphoenolpyruvate carboxykinase (PEPCK, 62 kDa, 1/1000) Santa Cruz, Dallas, TX, USA; peroxisome proliferator-activated receptor alpha (PPARα, 57 kDa, 1/1000) Cayman Chemical, Ann Arbor, MI, USA; phospho-AKT^Ser473^ (p-AKT, 60 kDa, 1/1000) ThermoFisher Scientific, p38 MAPK (43 kDa, 1/1000) ThermoFisher Scientific; sterol regulatory element-binding protein 1 (SREBP1c, 60 kDa,1/1000) Abcam; toll like receptor 4 (TLR4, 79 kDa, 1/1000) Abcam; TNF-alpha (TNF-α, 26 kDa, 1/1000) ThermoFisher Scientific; phospho-ACC (p-ACC, 257 kDa, 1/1000) Millipore; β-actin (43 kDa, 1/250,000) Sigma-Aldrich, Saint Louis, MO, USA. The relative amount of primary antibody was detected with a species-specific horseradish peroxidase-conjugated secondary antibody using a ChemiDoc MP Imaging System (Bio-Rad, Hercules, CA, USA). All data are expressed as the ratio of target protein to β-actin or GAPDH (used as housekeeping genes).

### 2.8. Statistical Analysis

All values are expressed as the mean ± SEM. Data were analyzed by one-way analysis of variance (ANOVA) followed by Tukey’s multiple comparisons test when three groups were compared. When the comparison was between two groups, a Student *t*-test was performed. Time points within different groups were compared using two-way repeated measures ANOVA with Student–Newman–Keuls post hoc test. PRISM 7.0 (GraphPad Software, San Diego, CA, USA) was used for statistical analysis. Differences were considered significant at *p* ≤ 0.05.

## 3. Results

### 3.1. Energy Intake, Body Weight and Body Composition of HFHS-Fed Mice Supplemented with GMP

**Figure 1:**
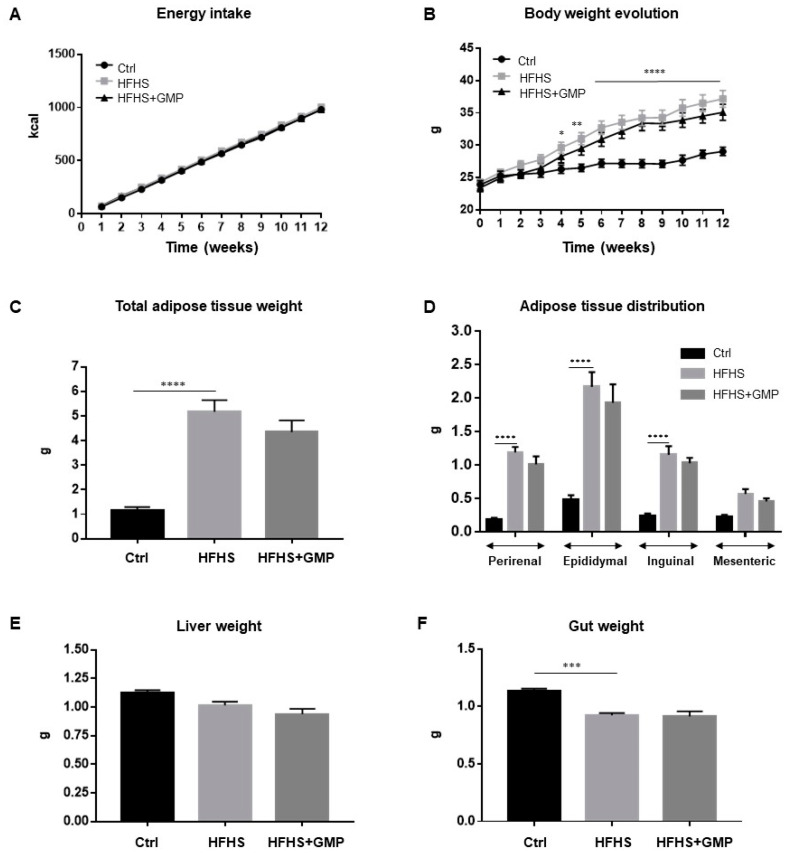
Impact of GMP on energy intake, body weight and body composition. Mice were fed either a standard chow diet (Ctrl) or a high-fat, high-sucrose diet (HFHS) for 12 weeks. HFHS-fed animals were treated with glycomacropeptide (GMP, 200 mg/kg body weight/day) by gavage (HFHS+GMP). Ctrl and HFHS-fed mice were gavaged with a water-vehicle. (**A**) Energy intake; (**B**) Body weight evolution; (**C**) Whole body adiposity; (**D**) Adipose tissue distribution; (**E**) Liver and (**F**) Gut weights were evaluated as described in Material & Methods. Data are expressed as the mean ± SEM for *n* = 10 mice/group. * *p* < 0.05, ** *p* < 0.01, *** *p* < 0.001, **** *p* < 0.0001 vs. Ctrl mice.

Mice fed a regular chow (Ctrl) or a HFHS diet did not differ in their energy intake (Figure 1A), but as expected, HFHS diet led to a significant increase in total body weight gain (Figure 1B). GMP supplementation did not protect from diet-induced obesity and fat deposition (Figure 1B,C). The elevated body weight gain in the HFHS group was associated with elevation of perirenal, epididymal, inguinal and mesenteric adiposity (Figure 1D), while remaining irresponsive to GMP treatment. No significant difference in liver weight was noted among groups (Figure 1E), whereas gut weight differed between Ctrl and HFHS mice (Figure 1F).

### 3.2. GMP Administration Attenuates Hyperinsulinemia, IR and Hypercholesterolemia in HFHS-Fed Mice

**Figure 2:**
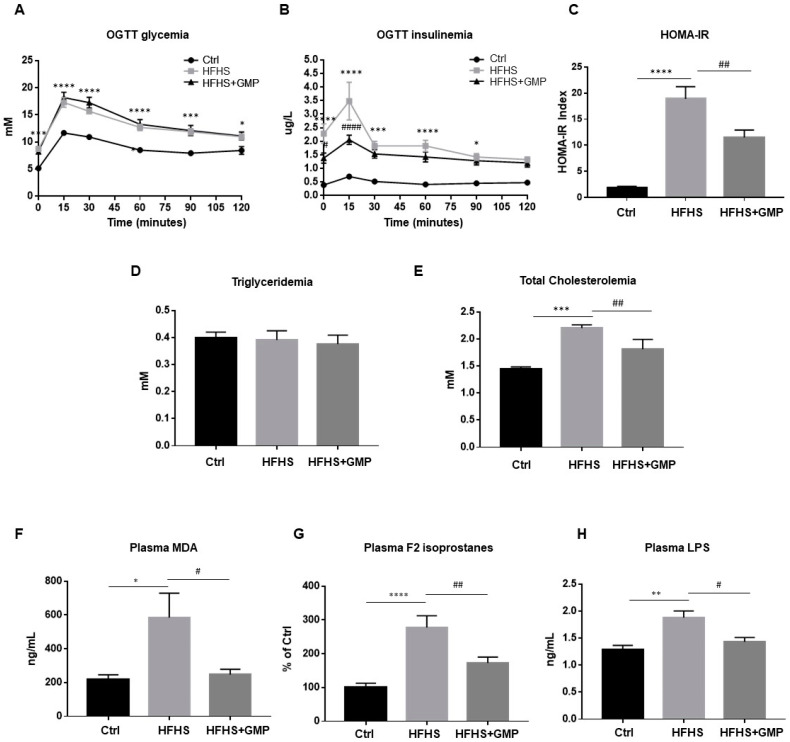
Effect of GMP administration on insulin resistance, dyslipidemia, metabolic endotoxemia as well as on systemic oxidative stress and inflammation in HFHS-fed mice. At week 10, after a 12 h overnight fast, chow (Ctrl), high-fat high-sucrose (HFHS), and HFHS-fed mice supplemented with glycomacropeptide (GMP) were subjected to an oral glucose tolerance test (OGTT). (**A**) glycemia and (**B**) insulinemia were measured as described in Material & Methods. (**C**) Homeostatic model assessment of insulin resistance (HOMA-IR) was calculated. At sacrifice, plasma (**D**) triglycerides, (**E**) total cholesterol, (**F**) Malondialdehyde (MDA), (**G**) F2 isoprostanes and (**H**) Lipopolysaccharide (LPS) concentrations were determined. Data are expressed as the mean ± SEM for *n* = 10 mice/group: * *p* < 0.05, ** *p* < 0.01, *** *p* < 0.001, **** *p* < 0.0001 vs. Ctrls; ^#^ *p* < 0.05, ^##^ *p* < 0.01, ^####^ *p* < 0.0001 vs. HFHS-fed mice.

At week 10, glucose homeostasis was examined by an OGTT (Figure 2A). Although GMP-HFHS-fed mice did not display improved glycemia during OGTT (Figure 2A), they exhibited lower plasma insulin levels (Figure 2B), suggesting an improved insulin sensitivity. This result was confirmed by the calculation of the HOMA-IR index, which also indicated that HFHS+GMP mice were less resistant to insulin (Figure 2C). At sacrifice, fasting plasma TG and TC levels were measured (Figure 2D,E). 

Triglyceridemia was not affected by HFHS diet nor by GMP treatment (Figure 2D). On the other hand, compared to Ctrls, HFHS animals displayed hypercholesterolemia, which was mitigated by GMP administration (Figure 2E). 

### 3.3. Improvement of Metabolic Endotoxemia as Well as Systemic Oxidative Stress and Inflammation by GMP

Given the implication of oxidative stress (OxS) in the occurrence and development of obesity and IR, we assessed lipid peroxidation by determining plasma levels of MDA and F2 isoprostanes, the latter being prostaglandin-like compounds. While HFHS diet promoted the formation of MDA and F2 isoprostanes compared to chow diet, GMP supplementation lowered their plasma levels (Figure 2F,G).

Metabolic endotoxemia has emerged as an important mediator in the pathogenesis of chronic low-grade inflammation, and plays a key role in the development of obesity and cardiometabolic disorders [32]. Therefore, we evaluated LPS levels in the three mice groups. Treatment of mice with HFHS enhanced plasma LPS concentrations compared to the chow group, whereas GMP supplementation produced its significant decrease (Figure 2H). In light of these findings, GMP is able to alleviate systemic OxS, inflammation, and metabolic endotoxemia in HFHS-induced obesity and IR.

### 3.4. GMP Impact on Hepatic Homeostasis 

**Figure 3:**
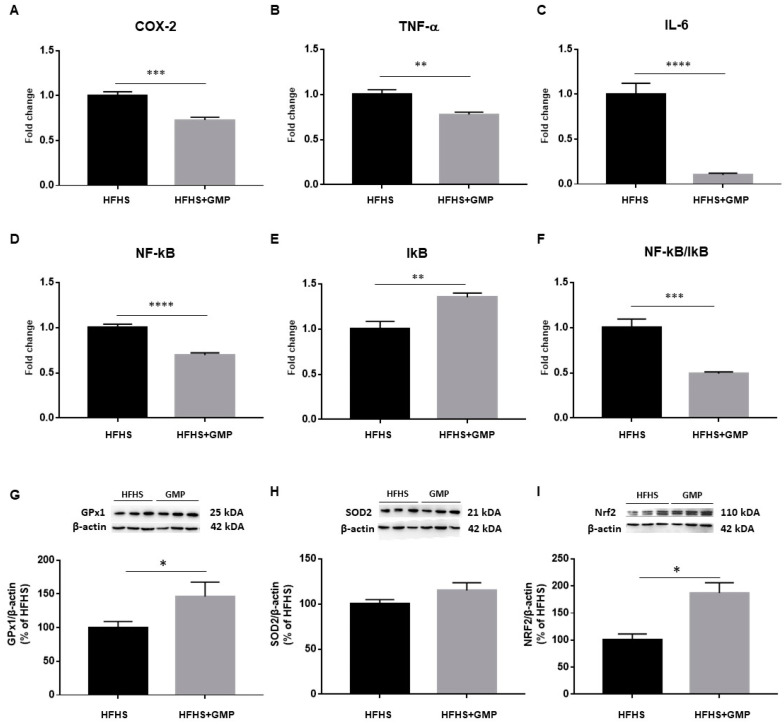
Anti-inflammatory and antioxidant effects of GMP in the liver of HFHS-treated mice. The gene expression of (**A**) cyclooxygenase-2 (COX-2), (**B**) tumor necrosis factor alpha (TNF-α), (**C**) interlukin-6 (IL-6), (**D**) nuclear factor-kappa B (NF-κB) and (**E**) inhibitor of kappa B (IκB) was determined by RT-qPCR as described in Section 2. (**F**) The NF-κB/IκB ratio was then calculated. The protein mass of antioxidant defense biomarkers: (**G**) glutathione peroxidase1 (GPx1), (**H**) superoxide dismutase 2 (SOD2) and the transcription factor (**I**) nuclear factor erythroid 2 related factor 2 (NRF2) was evaluated by Western blot as described in Material & Method section. Results represent the mean of *n* = 6–8 mice/group. * *p* < 0.05, ** *p* < 0.01, *** *p* < 0.001, **** *p* < 0.0001 vs. HFHS mice.

As an inflammatory state characterizes obesity and IR, we evaluated the gene expression of cyclooxygenase-2 (COX-2), TNF-α, IL-6, nuclear factor kappa B (NF-κB) and inhibitor of kappa B (IκB) by RT-qPCR in the liver (Figure 3A–E). The NF-κB/IκB ratio was also calculated (Figure 3F). The gene expression of all these hepatic inflammatory markers was significantly lower in GMP-treated compared to HFHS mice. To confirm the beneficial impact of GMP on diet-induced liver inflammatory mRNA markers, the expression of key proteins involved in inflammatory signaling pathways was assessed in the two mice groups by Western blot. Importantly, lower protein levels of TNF-α and IL-6 were observed in the HFHS+GMP group, corroborating the mRNA findings (Supplementary Materials, Figure S1). As LPS is known to induce the inflammatory cascade via TLR4 in the liver, we also assessed its protein expression, and noted a significant downregulation by GMP (Supplementary data). The protein expression of essential endogenous antioxidant biomarkers was next assessed, and GMP was found to upregulate GPx1 (Figure 3G) and NRF2 (Figure 3I) without altering SOD2 (Figure 3H). 

**Figure 4:**
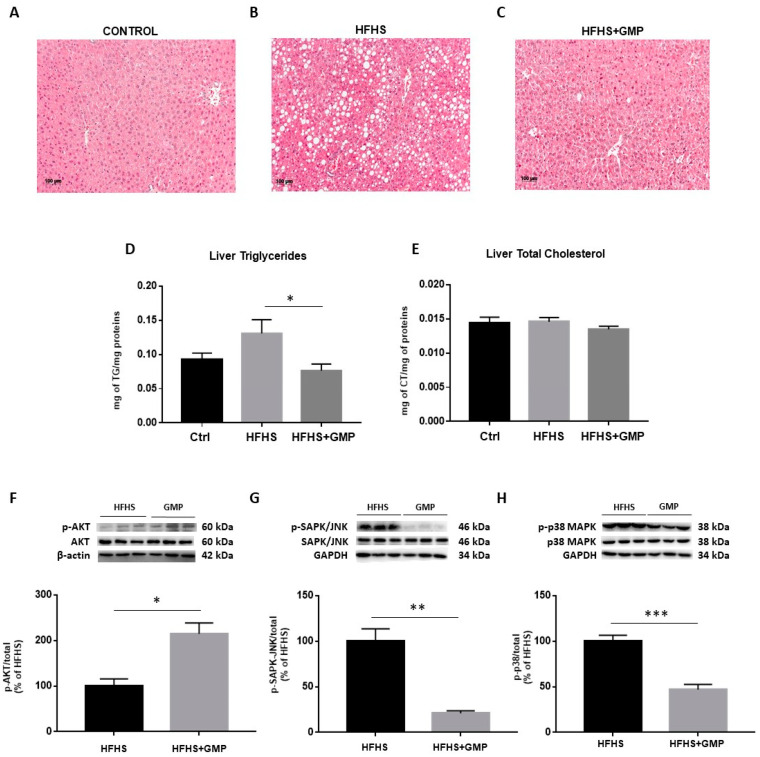
GMP reduces hepatic lipid accumulation and modulates insulin signaling pathway in HFHS-fed mice. Representative images of hematoxylin phloxine saffron stained liver sections of (**A**) chow- (Ctrl), (**B**) high-fat, high-sucrose (HFHS)- and (**C**) HFHS+GMP-fed mice; (**D**) Triglycerides and (**E**) total cholesterol content was quantified in liver of *n* = 10 mice/group; Protein expression of pivotal biomarkers influencing insulin sensitivity was determined in liver by Western blot as described in Material & Methods. (**F**) p-AKT/AKT, (**G**) p-SAPK/JNK/SAPK-JNK and (**H**) p-p38 MAPK/p38 MAPK ratios were calculated. Results represent the mean ± SEM for *n* = 3–6 mice/group. * *p* < 0.05, ** *p* < 0.01, *** *p* < 0.001 vs. HFHS-fed mice.

Since HFHS diet triggers hepatic lipid accumulation, liver fat deposition was assessed. Histopathological HPS staining revealed marked differences in the number of lipid droplets between Ctrls (Figure 4A), HFHS (Figure 4B) and HFHS+GMP (Figure 4C) mice. HFHS mice presented with micro, macro or a combination of both, whereas mice in the HFHS+GMP group displayed lessened severity of fat deposition (Supplementary Materials, Figure S2). In fact, hepatic lipid accumulation in GMP-treated animals was closer to Ctrls than HFHS animals (Supplementary Materials, Figure S2). Subsequently, GMP decreased liver TG (Figure 4D), but not TC content in HFHS+GMP mice (Figure 4E). 

Given the close association between hepatic lipid accumulation and IR, we determined the protein expression of AKT, a driving force for the activation of key metabolic events. While no change was detected in the protein expression of total AKT, its phosphorylation was enhanced by GMP (Supplementary Materials, Figure S3) and led to elevated p-AKT/AKT ratio in the livers of HFHS+GMP mice (Figure 4F). As insulin signaling is known to be affected by MAPK activity, especially p38 MAPK and SAPK/JNK, we investigated their protein expression. While no difference was detected in total SAPK/JNK and p38 MAPK protein expression between groups, their phosphorylation status (Supplementary Materials, Figure S3) was lessened by GMP, leading to lower p-SAPK/JNK/SAPK/JNK (Figure 4G) and p-p38 MAPK/p38 MAPK ratios in HFHS+GMP group (Figure 4H).

**Figure 5:**
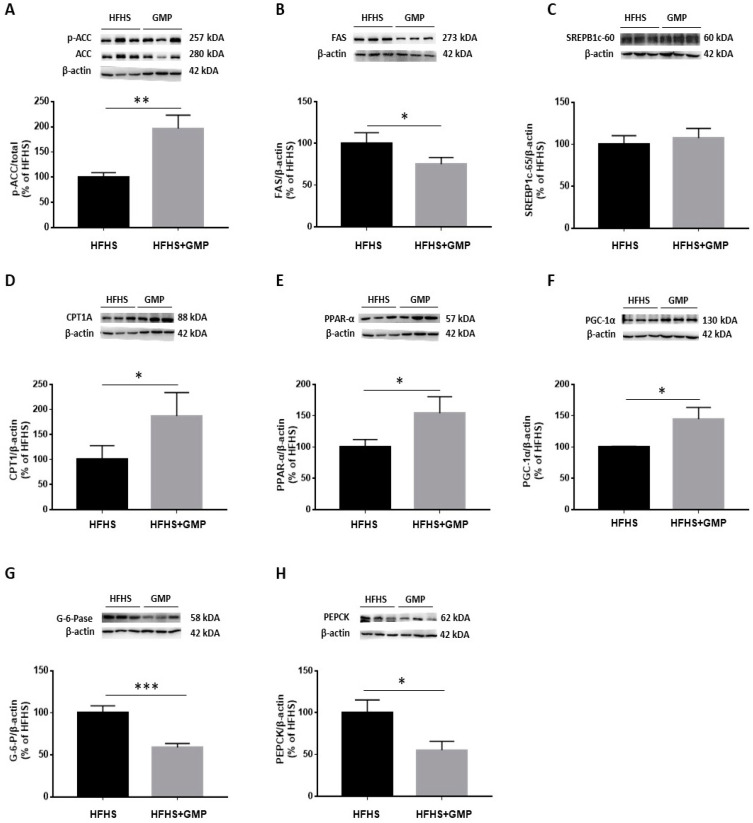
GMP modulates lipid and glucose metabolism in liver. (**A**) p-ACC/ACC ratio was calculated after analysis of protein expression of ACC and p-ACC, as well as (**B**) FAS, (**C**) SREBP1c, (**D**) CPT1A, (**E**) PPAR-α, (**F**) PGC-1α, (**G**) G-6-Pase and (**H**) PEPCK by Western blot. Data are expressed as the mean ± SEM for *n* = 3–6 mice/group. * *p* < 0.05, ** *p* < 0.01, *** *p* < 0.001 vs. HFHS mice.

The reduced amounts of lipid droplets found in the livers of GMP treated animals prompted us to evaluate the mechanisms of action leading to hepatic steatosis. In a first step, we assessed the protein expression of critical lipogenic enzymes. Western blot analysis clearly showed an augmentation of p-ACC/ACC ratio (Figure 5A). As ACC phosphorylation restricts ACC activity, this finding suggests that GMP lowered the carboxylation of acetyl-CoA to produce malonyl-CoA. Accordingly, a decline was noted in FAS, an enzyme with a pivotal role in de novo lipogenesis in response to GMP treatment (Figure 5B). 

We next assessed the protein expression of SREBP1c, an important transcription factor involved in lipid metabolism. Surprisingly, no difference was noted in SREBP1c protein mass (Figure 5C) although GMP lowered its gene expression (Supplementary Materials, Figure S4). 

Next, we evaluated the expression of CPT1A, which represents a key mitochondrial enzyme involved in fatty acid (FA) β-oxidation. We found that GMP supplementation significantly increased its protein expression (Figure 5D). Thereafter, we determined the protein mass of PGC1-α and PPAR-α, two central transcription factors regulating mitochondrial function/biogenesis, and intracellular lipid metabolism. GMP significantly raised PPAR-α (Figure 5E) and PGC1-α protein expression (Figure 5F). These observations suggest that GMP prevented lipid accumulation in the liver by promoting the catabolism of mitochondrial FAs. Excessive stimulation of enzymes involved in the regulation of gluconeogenesis contributes to exaggerated hepatic glucose production, and consequently, to hyperglycemia. We assessed the protein expression of two crucial enzymes involved in these processes: PEPCK and G-6-Pase. We found that their protein expression was significantly lowered in the HFHS+GMP compared to the HFHS group (Figure 5G,H).

## 4. Discussion

The present work highlights the beneficial modulatory effects of GMP, a bioactive milk peptide, on systemic and liver abnormalities induced by a HFHS diet in mice. In fact, supplementation with GMP (200 mg/kg body weight/day) led to improvement of systemic IR and hepatic insulin sensitivity. Furthermore, GMP mitigated HFHS mediated-hepatic steatosis, OxS and inflammation, as well as lipogenesis and gluconeogenesis while enhancing FA β-oxidation, in association with metabolic endotoxemia normalization. Therefore, in this preclinical study, bioactive bovine milk-derived GMP displays a wide range of health-promoting metabolic functions.

Given the persistent prevalence of MetS worldwide and the use of multiple pharmacological agents to treat its various harmful components, there is an urgent need to test non-pharmaceutical nutraceuticals able to target several MetS features. In this context, mice were fed an enriched HFHS diet for 12 weeks to induce metabolic dysfunctions, with a view of examining their alleviation by a daily GMP intake. This diet caused obesity, hyperglycemia, IR, metabolic endotoxemia and hypercholesterolemia, all known as important MetS components. Nevertheless, HFHS diet administration did not result in hypertriglyceridemia, which is in line with previous studies [33,34,35]. It is interesting to mention the observations from a recent report, which suggest that thermoneutral housing at 30–32 °C is more adequate to recapitulate human noncommunicable metabolic diseases than the standard pervasive temperature (22 ± 1 °C) [36].

Our initial approach was to assess the influence of GMP on IR as the latter plays a central role in interlinking the various constituents of MetS [37]. GMP significantly lowered HOMA-IR index, which usually marks for both the presence and extent of IR [38]. As in the IR condition, peripheral organs fail to respond to insulin, we also examined liver insulin sensitivity in HFHS-fed mice. Specifically, we looked at the AKT signaling pathway, which mediates the major biological effects of insulin in the cell [39]. Since decrease in its activity directly results in IR [40,41]. GMP ability in stimulating the hepatic AKT insulin signaling pathway suggests greater insulin sensitivity. As reported in our recent review [10], very little attention has so far been paid to the effectiveness of GMP, one of the most biologically active milk components [42], to restore insulin sensitivity in cardiometabolic disorders. Therefore, our original data are a good start in the global evaluation of the function of GMP in preventing and treating IR.

The second step was to estimate the potential of GMP to alleviate OxS and inflammation, which uphold IR. Clearly, GMP showed capacity in fighting OxS in the circulation (MDA and F2 isoprostanes) and liver (GPx1), and in alleviating liver inflammation, which is documented by a decline in inflammatory biomarkers (COX-2, TNF-α and IL-6). To understand the mechanisms of action, we evaluated the expression of two powerful rapid acting transcription factors, NRF2 and NF-κB, the former promoting the antioxidant defense system, and the last upregulating inflammatory agents [43]. Our results showed that GMP activated NRF2 (resulting in a rise in GPx1, a downstream phase II enzyme) while lowering NF-κB, leading to the decline of inflammatory components. 

Diet-induced metabolic endotoxemia triggers the development of many chronic inflammatory conditions such as MetS and NAFLD [44,45]. We therefore measured circulating LPS levels and evaluated fat accumulation in the liver. Our observations evidenced a marked elevation of plasma LPS, histologically fat deposition in the liver, and biochemically hepatic TG augmentation in response to a 12 week-HFHS feeding. However, GMP supplementation mitigated these abnormalities in association with inflammation relief, as demonstrated by downregulation of TLR4/NF-κB signaling pathway. Our data suggest an action of GMP on gut-liver axis given the strong link between gut microbiota-derived LPS, intestinal permeability and the NAFLD pathogenesis [46]. Although further studies are needed to validate this assumption, it seems reasonable to propose it since GMP was shown to exert anti-adhesive microbial properties, to bind to pathogens responsible for severe intestinal infections, and to display bifidogenic activity [10]. In light of our beneficial observations on liver steatosis and complications in response to GMP in the MetS context, it will be judicious to investigate the role of this nutrient in NAFLD pathogenesis using appropriate NAFLD animal models [47].

The close interaction between intestinal and hepatic tissues in normal physiology and disease [48], and the indication that the liver is an appealing target for translocation of gut microbes and their products (e.g., LPS) prompted us to investigate the metabolic status of the liver. In line with the reduced lipid deposition found in the livers of GMP-treated animals, there seemed to be a decreased lipogenesis according to the lowered expression of FAS and ACC enzymes. Another outcome of our study is the refurbishment of the FA β-oxidation pathway by GMP via upregulation of the rate-limiting CPT1A factor, which contributed to lessen HFHS-induced fat deposition in the liver. Our findings showed that the mechanisms of action of GMP are via the induction of PPAR-α (capable to stimulate the transcription of CPT1A). Importantly, GMP promoted PGC-1α that positively regulates insulin sensitivity, mitochondrial biogenesis and lipid oxidation [49,50]. It is very likely that the substantiative modulation of FA β-oxidation by GMP resulted through docking and coactivation of PGC-1α on PPAR-α, which is the frequent PGC-1α form of action in regulating diverse genes of the electron transport chain, mitochondrial biogenesis and FA β-oxidation [50].

Striking differences exist between men and women in NAFLD pathobiology [51]. As a matter of fact, 13.2% of men versus 8.7% of women are affected by NAFLD worldwide [52]. The evidence that prevalence of NAFLD tends to increase in women after menopause suggests a protective effect of estrogen [51]. Indeed, results from numerous animal studies show that estrogen is protective against hepatic lipid accumulation and inflammation [53,54,55]. As the prevalence and severity is higher in men than in women [51,52], we first decided to study GMP impact on liver dysmetabolism in male animals. However, given the importance of sex differences in NAFLD and the research gap in the literature [51], further investigation is needed to determine GMP specific impact on hepatic lipid accumulation and metabolism in female animals.

As insulin mediated suppression of hepatic gluconeogenesis is altered in MetS and type 2 diabetes mellitus [56,57], we investigated the effectiveness of GMP to bring this pathway to normality in obesogenic conditions. We specifically focused on PEPCK and G-6-Pase, two critical enzymes able to convert non-sugar substances into glucose, since their upregulation is tightly associated to raised gluconeogenesis and explains why these crucial enzymes serve as targets for diabetes treatment [58]. Our experiments revealed the downregulation of PEPCK and G-6-Pase by GMP, which emphasizes its anti-gluconeogenesis action. It is worth mentioning the recent evidence documenting the reciprocal relationship between PEPCK/G-6-Pase expression and AKT phosphorylation [59]. Our findings show reinforcement of AKT phosphorylation in response to GMP in HFHS conditions, thereby suggesting interconnected control of insulin sensitivity and gluconeogenic programme by the nutritional GMP factor. It is important to underline that IR-mediated prooxidant-antioxidant imbalance and raised inflammation is intimately linked to the intrahepatic alterations in de novo lipogenesis, FA β-oxidation and gluconeogenesis [60].

In conclusion, the present investigation shows that GMP supplementation is negatively associated with OxS, inflammation and IR. Furthermore, GMP can provide liver protection against metabolic endotoxemia and obesogenic diet damage. In particular, the mechanisms of GMP are through its antioxidant, anti-inflammatory and insulin sensitizing actions, as well as via regulation of lipogenesis, FA β-oxidation and gluconeogenesis through powerful transcription factors.

## Data Availability

Not applicable.

## Supplementary Materials

The following are available online at https://www.mdpi.com/article/10.3390/biomedicines9091140/s1, Table S1: List of primers used for RT-qPCR analysis; Figure S1: Glycomacropeptide supplementation attenuates diet-induced liver inflammation; Figure S2: Hepatic lipid accumulation is attenuated in high-fat, high-sucrose-fed mice supplemented with glycomacropeptide; Figure S3: Glycomacropeptide increases hepatic insulin sensitivity, downregulates mitogen-activated protein kinases, and modulates lipid metabolism in high-fat, high-sucrose-fed mice; Figure S4: SREB1c gene regulation by glycomacropeptide.
