# Supplementary material for: Glycomacropeptide for Management of Insulin Resistance and Liver Metabolic Perturbations
Source: Biomedicines. 2021 Sep 2;9(9):1140. doi: 10.3390/biomedicines9091140 (PMC8469639; doi:10.3390/biomedicines9091140)
Supplement: Supplementary file 1 [file biomedicines-09-01140-s001.zip › biomedicines-1352368-supplementary.pdf]

Supplementary Table

Table S1. List of primers used for RT-qPCR analysis.

	Primers
**Cox-2**	Forward : GCTGTACAAGCAGTGGCAAA
	Reverse : CCCCAAAGATAGCATCTGGA
**TNFα**	Forward : GAACTGGCAGAAGAGGCACT
	Reverse : AGGGTCTGGGCCATAGAACT
**IL-6**	Forward : GTTCTCTGGGAAATCGTGGA
	Reverse : TTCTGCAAGTGCATCAT
**NFκB**	Forward : AGCTTCACTCGGAGACTGGA
	Reverse : ACGATTTTCAGGTTGGATGC
**IκB**	Forward : TGGCCAGTGTAGGCAGTCTTG
	Reverse : GACACGTGTGGCCATTGTAG
**Srebp1c**	Forward : GACCCTACGAAGTGCACACA
	Reverse : TCATGCCCTCCATAGACACA

Supplementary Figures

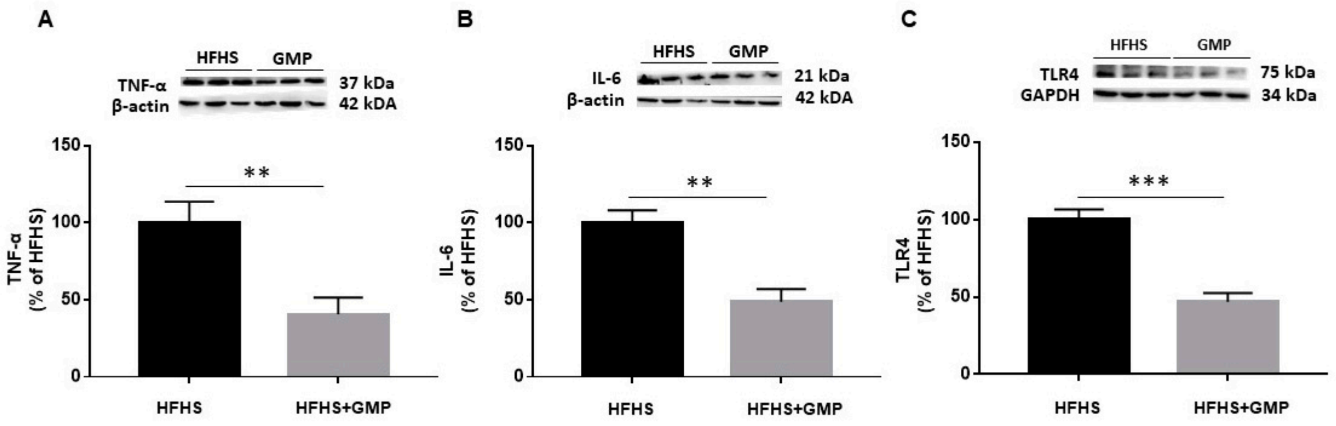

Figure S1. Glycomacropeptide supplementation attenuates diet-induced liver inflammation. Protein levels of inflammation biomarkers were evaluated by Western blot in livers of high-fat high-sucrose (HFHS), and HFHS-fed mice supplemented with glycomacropeptide (GMP): (A) tumor necrosis alpha (TNF-α), (B) interlukin-6 (IL- 6), and (C) toll like Receptor 4 (TLR4). Data are expressed as the mean ± SEM of 3-6 mice in each group ** P <0.01, *** P <0.001 vs. HFHS

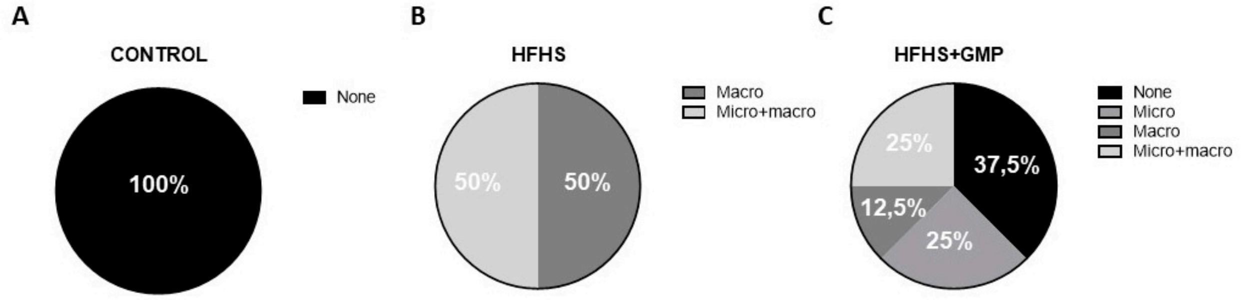

Figure S2. Hepatic lipid accumulation is attenuated in high-fat, high-sucrose-fed mice supplemented with glycomacropeptide. From the images of hematoxylin phloxine saffron (HPS) stained liver sections of chow-fed mice (Ctrl), high-fat, high-sucrose (HFHS)-fed mice, and HFHS-fed mice supplemented with glycomacropeptide (GMP), percentages of microvesicular or macrovesicular steatosis were determined calculating the steatotic fraction area relative to the entire tissue area. The graphs summarize the presence of microsteatosis (micro), macrosteatosis (macro) or the combination of both (micro+macro) in each group (n = 8): (A) controls, (B) HFHS, and (C) HFHS+GMP.

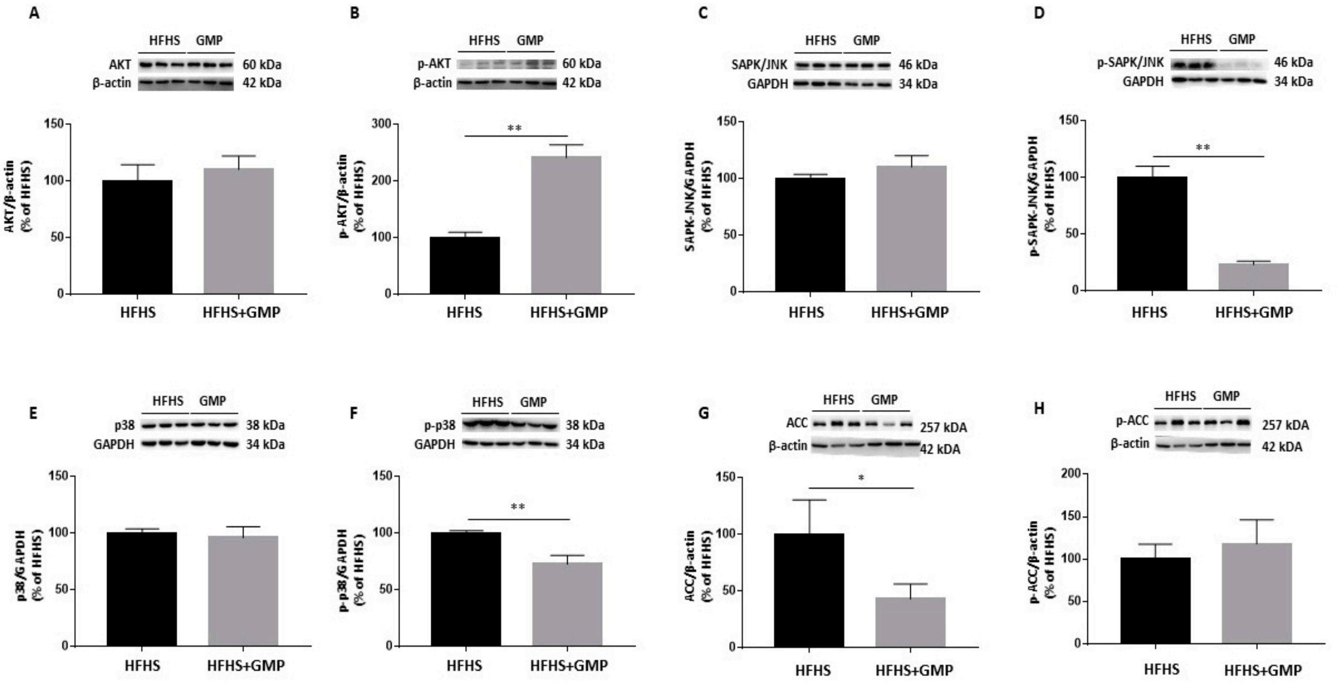

Figure S3. Glycomacropeptide increases hepatic insulin sensitivity, downregulates mitogen-activated protein kinases and modulates lipid metabolism in high-fat, high-sucrose-fed mice. Protein expression of the following important biomarkers was determined by Western blot in livers of high-fat high-sucrose (HFHS), and HFHS-fed mice supplemented with glycomacropeptide (GMP): (A) AKT; (B) phospho AKT, (C) stress-activated protein kinase (SAPK)/Jun amino-terminal kinases (JNK), (D) phospho SAPK/JNK, (E) p38-mitogen activated protein kinase (MAPK), (F) phospho p38, (G) acetyl CoA carboxylase (ACC), and (H) phospho ACC. Results represent the mean ± SEM of 3-6 mice in each group. ** *P* <0.01 vs. HFHS

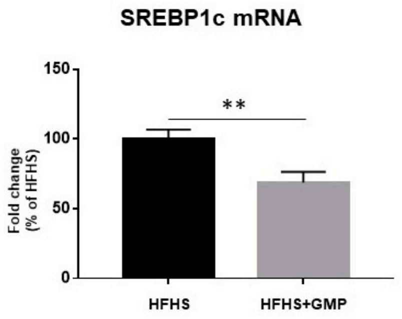

Figure S4. SREB1c gene expression regulation by glycomacropeptide. Gene expression of SREBP1c was assessed by RT-qPCR in HFHS and HFHS+GMP groups. Results represent the mean ± SEM of 6 mice in each group. ** P <0.01
